# Let’s DAG in: how directed acyclic graphs can help behavioural ecology be more transparent

**DOI:** 10.1098/rspb.2025.0963

**Published:** 2025-07-23

**Authors:** Mirjam J. Borger, Aparajitha Ramesh

**Affiliations:** ^1^ Department of Evolutionary Biology, Bielefeld University, Bielefeld, Nordrhein-Westfalen, Germany; ^2^ Department of Behavioural Ecology, Institute of Ecology and Evolution, University of Bern, Bern, Switzerland

**Keywords:** causal inference, science communication, bad controls, reproducibility, best practices

## Abstract

Directed acyclic graphs (DAGs) are powerful tools for visualizing assumptions/hypotheses and causal inference. Although their use is becoming more widespread across various disciplines, they remain underutilised in behavioural ecology and evolution. Here, we point out why DAGs can serve as highly valuable tools in this field, particularly in the context of observational and field studies, which can feature many variables with complex relationships. Using concrete examples, we show that including DAGs in empirical studies helps clarify and summarize the key underlying assumptions, which are often implicit. With that, DAGs can be used to make researchers aware of bad controls and help them to explicitly think through the relationship between variables and their inclusion in statistical models. In addition, providing DAGs makes the work of reviewers and meta-analysis researchers easier, more rigorous and reliable. Overall, DAGs enhance understanding and transparency, ultimately improving study reproducibility and contributing to greater reliability and replicability across the field. With this paper, we hope to encourage all behavioural ecologists to include DAGs in their papers.

## 1. Introduction

Directed acyclic graphs (DAGs) are graphical models to visualize the different variables and their assumed effects on each other within a study system. Indeed, DAGs are graphical representations of your hypothesis and form the cornerstone of your statistical model, by formalizing the causal structure between variables underlying the hypothesis. DAGs represent variables as nodes, connected by arrows pointing towards assumed causal effects (‘directed’; see §1*c* for an explanation of some important terminology and usage). Crucially, DAGs are non-circular (‘acyclic’), that is, cause and effects do not feed back, and thus an assumption has to be made about which variable is the cause and which one is the consequence for the specific circumstance that is being studied. DAGs are well-established tools of causal inference and their use is increasing across different fields (e.g. in econometrics [1]; environmental sciences and ecology [2,3]; epidemiology and clinical studies [4–6]), but they are sparsely used in others (such as behavioural ecology). Here, we aim to demonstrate that DAGs not only provide a robust framework for statistical analyses but also enhance transparency and replicability in research. Moreover, by visualizing and comparing different DAGs across various systems for similar questions, we can scrutinize the underlying causal structures, offering new insights and potentially driving innovative inquiries in behavioural ecology. Thus, we argue that there are more benefits to using DAGs in research, other than their role of formalizing statistical models and avoiding common pitfalls (such as the inclusion of colliders or pipe variables). In order to encourage their incorporation, we also discuss how to get started by reviewing key terminology and causal structures (see §2, feel free to skip this section when you are already familiar with causal inference), while also guiding researchers to essential literature.

### (a) Is this not already known?

DAGs are already a well-established concept (e.g. [7–9]), playing a central role in causal inference. They frequently accompany studies employing path analyses or structural equation models. Yet, DAGs remain underutilised in biology and especially in behavioural ecology. To demonstrate this point, we reviewed six issues of the journal *Behavioral Ecology* (*n* = 123 original articles, volume 34, issues 4−6 and volume 35, issues 1−3), and six volumes of the journal *Animal Behaviour* (*n* = 122 research articles, volumes 210−215), and found that only one article contained a DAG (see our data and analysis [10]). This sample includes some articles that might not benefit from the use of a DAG (e.g. theoretical biology papers), but most of these papers included a statistical analysis of empirical data that might benefit from the inclusion of a DAG, as we will argue in the following sections.

### (b) Current practices

Statistical modelling is generally performed for one of the two main reasons: prediction or causation [9]. For example, prediction applies when trying to extrapolate population size of a conserved species in the next years based on current information, or when inferring how fast a virus will spread in a population; whereas causation applies to performing an experiment to infer if *x* causes *y* and if this is a small or large effect, or by trying to find associations between variables, which ultimately means that *x* and *y* are directly or indirectly causally linked in some way, to understand if their relation is worthy of further study. Most statistical models are optimized for the first goal (‘the best-fitting model’), despite being commonly used for addressing the second [11]. Behavioural ecology is a field that mostly focusses on the second goal [12,13]. This is evident from the use of phrases such as ‘*x* increases performance of *y*’ or ‘*x* seems to be driven by *y*’ [12]. Articles often verbally describe causal models and assumptions in §1, leading to the main question or hypothesis, and to a certain extent these causal models and assumptions are further described in the methods. Using a formal mathematical equation, structural causal models (SCMs) or tools other than DAGs to express underlying causal assumptions or structures are not the norm. In many cases, causal language is vague as researchers keenly identify that correlations are not causations in the absence of randomized controlled experiments [12,14], but the overall goal still remains to learn something about causation. This is often reflected in a vaguely causal discussion section, which then acts as a starting point for researchers following up on similar topics, essentially leading to causal assumptions that are not coherent or principled. Moreover, in the methods, it is often mentioned that additional variables are added to models as a ‘control’ in the analysis, while these variables are often not mentioned in the hypothesis, and their inclusion is often not justified statistically (e.g. ‘we controlled for *z* in the analysis as we expect *z* to affect *y* or we controlled for *z* because we expect that *z* is a confounder of *x* and *y*). Some articles describe that only uncorrelated variables are added to models, but again often no justification for this addition is given (e.g. ‘we were also interested in the effect of *z* on *y*, so we also included this in the model’ or ‘we wanted to increase the precision of the estimate of the effect of *x* on *y*, and therefore we have included *z* in our model’). Similarly, variables are often later omitted from the statistical models citing collinearity. Hence, although researchers think about their assumptions and hypotheses when performing their analyses, this is often vaguely explained and not principled when it comes to their actual analyses.

## 2. The main ingredients of DAGs

In this part, we provide some terminology and fundamentals that are crucial for beginning to use DAGs and understanding the literature on causal inference using DAGs. This part can be skipped if readers are familiar with it.

DAGs are essentially composed of nodes, represented by circles, which are the variables and directed, or single headed, arrows depicting a causal relationship.

### (a) Terminology

(1) Estimand: the target quantity that is to be estimated in an analysis. This is related directly to our research question and is what we aim to calculate in our statistical analysis. In our simple examples below, the estimand is the direct effect of *X* on *Y*.
(2) Direct effect: an effect that the change of a particular variable of interest (*X*) has on the outcome variable (*Y*). This means that we are only interested in the arrow that leads directly from *X* to *Y* and not the other arrows that emerge from *X* via another mediator to Y. This is achieved by blocking the other pathways that are not direct (see point 5 below).
(3) Total effect: the effect of our variable of interest (*X*) on our response variable (*Y*) via all the direct and indirect paths, but excluding the effects due to common causes or confounders (see §2b).
(4) Conditioning: also referred to as ‘controlling’, ‘adjusting’, ‘stratifying’ or ‘partialling out’ an effect, conditioning refers to the isolation of effects of the variable(s) of interest (*X*) on the outcome variable (*Y*) for a given value of the conditioned variable. This is often carried out by including the variable to condition as a covariate in the statistical model.
(5) Blocking: blocking a pathway means blocking a causal effect and therefore association among variables via that path. In the case of a confounder, we want to block the causal effect of the confounder by conditioning on it, while in the case of a collider (see §2b), the causal pathway is blocked when it is not conditioned on. Furthermore, in experimental designs, blocking can be achieved by randomization. For example, the effect of ‘time of day’ on an experimental outcome can be blocked by randomizing trials over the day or performing trials only at fixed time points in a day.

### (b) Fundamental causal structures

**Figure 1.**
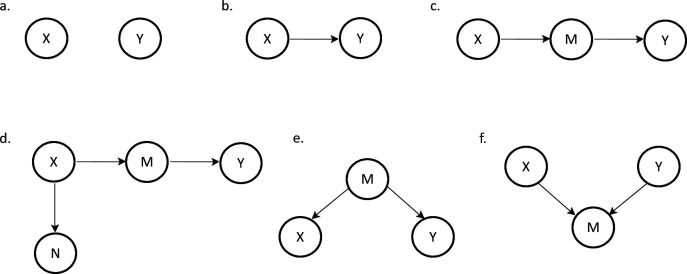
Fundamental causal structures. (a) Total independence. (b) Dependence: *X* causes *Y*. (c) Pipe or chain. (d) Descendant. (e) Confounder. (f) Collider.

Here, we will briefly touch upon the fundamental structures used in DAGs (figure 1). In these examples, we are interested in the direct effect of *X* on *Y* (the estimand). We can represent this by *Y*~*X*. When we want to test this in our generalized linear (mixed) models in *R*, we would write this as glm(*y*~*x*), which is the simplest model. For each of these cases, we will show the relationship between *X* and *Y* as a DAG. We here give examples of model statements in *R*, for GLM, as we would specify in the lme4 package [15]. Note that, we are showing the basic model structure as we use in *R*, without random effects or assumptions on the distribution of variables.

(a) Total independence: *X* and *Y* are independent of each other and have separate underlying causal variables. Therefore, variation in *X* is independent of variation in *Y*. See figure 1a.
(b) Dependence: *X* causes *Y*, or a change in the value of *X* causes a change in the value of *Y*. This takes the usual statistical model form, glm(*y*~*x*). Note that this need not necessarily be a linear relationship. See figure 1b.
(c) Pipe/Chain: pipes are variables that are caused by *x* and are causing *y*. Pipes do not necessarily have to be one variable but can also be a chain of variables. In figure 1c, *M* mediates the effect of *X* on *Y*. Conditioning on *M* leads to blocking of the causal pathway, making *X* and *Y* independent. That is, if we do glm(*y*~*m*+ *x*), we will not find a relationship between *x* and *y* anymore. In this case, there are no direct effects of *X* on *Y*, but there is a total effect of *X* on *Y* mediated via *M*.
(d) Descendant: when *X* causes *M*, *M* is also termed descendant of *X* and *X* is the ancestor of M. Similarly, in figure 1d, N is also a descendant of *X* and *Y* is a descendant of *M*. Descendants are important because conditioning on descendants of a variable can have a similar effect as conditioning on the variable, depending on the strength of their relationships. If we condition on *N* by glm(*y*^~^*x*+ *n*), we may only partially uncover or not uncover the effect of *X* on *Y*. This property may be particularly crucial to consider when descendants are present and measured, but the ancestor is not measured, in the case of collider or confounder causal structures (described below).
(e) Fork/confounder: forks or confounders are variables that cause both *X* and *Y*. In figure 1e, *M* is the confounder, which is the common causal ancestor that affects both *X* and *Y*, leading to a correlation. Conditioning on *M* leads to independence of *X* and *Y*. glm(*y*~*x*+ *m*) is the correct form for this causal structure. In some cases, when a confounder variable is unobservable but a descendant of the confounder was measured, conditioning on the descendant is important to at least partially control for the effects of the confounder on *X* and *Y*, to avoid a confounding relationship between *X* and *Y*.
(f) Collider: colliders are variables that are caused by both *X* and *Y*. In figure 1f, *M* is not the mediator but the common causal descendant of both *X* and *Y*. The causal path between *X* and *Y* are closed as long as *M* is not conditioned on. When a collider is added to a statistical model, a relationship between *X* and *Y* will be found (the causal path that leads through the collider variable). Hence, in this case, the correct model takes the form of glm(*y*~*x*). In cases where a collider has a descendant, it is also important not to condition on this descendant. Conditioning on a descendant of a collider will have the same effect of conditioning on the collider, depending on the strength of their relationships.

For a tutorial on the basic causal structures in *R*, we refer to the blog post by Peder M. Isager [16].

### (c) Example

**Figure 2.**
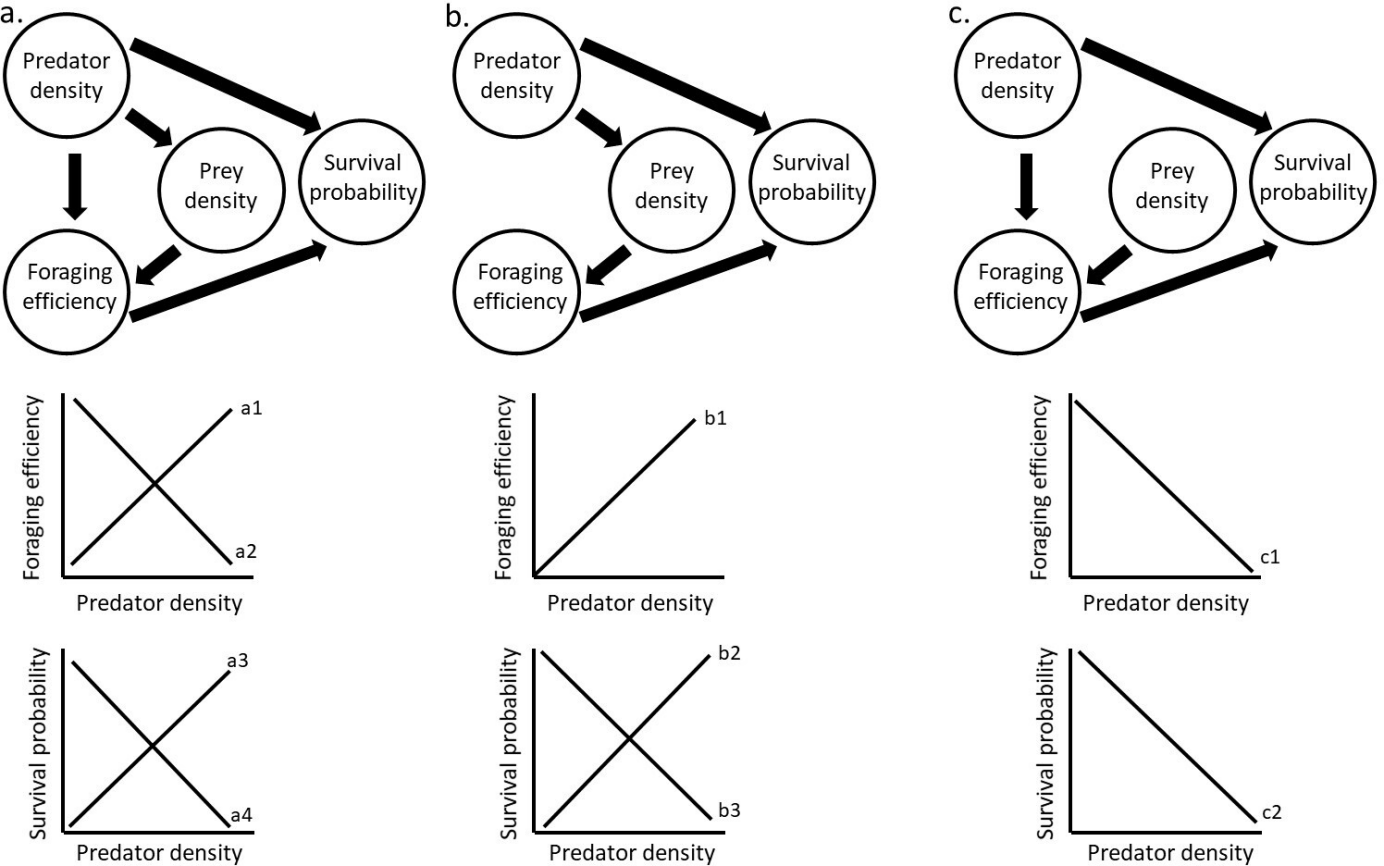
Three directed acyclic graphs about the effects of predator density, prey density and foraging efficiency of prey on the survival probability of prey individuals. (a) Causal structure where predator density both directly and indirectly affects foraging efficiency, and directly and indirectly affects the survival probability of prey. (b) Same causal structure but without a direct effect of predation on foraging efficiency (e.g. a nocturnal predator), and (c) The same causal structure as (a), but without a direct effect of predator density on prey density (e.g. a very large prey population). (a1)–(c2) represent the total effects that predator density could have on foraging efficiency and prey survival probability. In some cases, it is not possible to have a clear prediction and anything from a positive to negative relationship could be expected (e.g. (a1) and (a2)), while in other cases, it is possible to have a prediction (e.g. (b1)). Whether it is possible to have a prediction about a total effect depends on the strength and direction of each of the direct and indirect effects that together cause the total effect. Predicting the direction of a total effect can be challenging, especially if (in)direct effects are expected to have opposite effects.


Now, let us take the example DAG in figure 2a. The question we are interested in is the direct effect of predator density on prey survival probability, which is our estimand. Our *X* is predator density and *Y* is survival probability. If we want to look at the direct effects of predator density on survival probability, without the effect it may have on foraging efficiency, we need to condition on foraging efficiency, as this is a pipe variable. This will block all causal paths going to and emerging from foraging efficiency. It is important to note that labelling a variable as a confounder or a collider is always relative to the estimand and the structure of causal pathways between the predictor and the response variable. In this case, our model would look like

(2.1) glm(Survivalprobability ∼ Predatordensity +Foragingefficiency).

Predators reduce prey densities, leading to more efficient foraging and therefore increasing survival probability due to larger size, better health, etc. Therefore, if we are interested in the whole ecological picture, we are interested in the total effect of predator density on survival probability (our new estimand). To achieve this, we should not condition on any variables, allowing all causal pathways (both direct and indirect paths) between predator density and survival probability to be captured in a single estimate. This includes three pathways:

‘Predator density’ → ‘Prey density’ → ‘Foraging efficiency’ → ‘Survival probability’

‘Predator density’ → ‘Foraging efficiency’ → ‘Survival probability’

‘Predator density’ → ‘Survival probability’

And our model in this case would look like:


(2.2) glm(Survivalprobability ∼ Predatordensity )

## 3. Why should we use DAGs?

DAGs have two main benefits in empirical biology research. First, DAGs have been well-established as justifications of statistical models (e.g. regression models, which are most commonly used) and enable us to take a principled approach to our analyses [8,9]. Even without mastering the mathematics and theory behind DAGs and causal inference, we can translate our knowledge about the system into a DAG and benefit from the rather simple rules of DAGs to understand which variables should be included in a statistical model and how to overcome potential biases. We will give a short overview of the use of DAGs for statistics in the context of behavioural ecology, and explain what bad controls are and that they are largely unknown or neglected in this field. Second, DAGs can increase the transparency, readability and effectiveness of science communication, which could contribute to solving the replication crisis.

### (a) DAGs as justification for statistical models

DAGs have generally been used to justify which (control) variables should be included in or excluded from statistical models. Yet, so far, there seems to have been little notice of the concept of bad controls in behavioural ecology [17,18]. In our review of the 245 research articles, we found that predominantly, variables were included ‘to control for them’ without specifying why this was necessary. The common justification given for control variables to be included in a model is ‘biological relevance’. While biological relevance is an important criterion (why include something in a statistical model that is irrelevant for the response variable), it is not a clear justification for inclusion in a statistical model without considering the underlying causal assumptions. Especially in long-term study systems where many variables are measured over a long time, it can become tempting to add control variables without considering how this affects the estimated effect in question. In some cases, the addition of control variables can do harm rather than good, by falsely changing the estimate of the relation in question, while in other cases, controlling for a variable is necessary to obtain a correct estimate of an effect.

**Figure 3.**
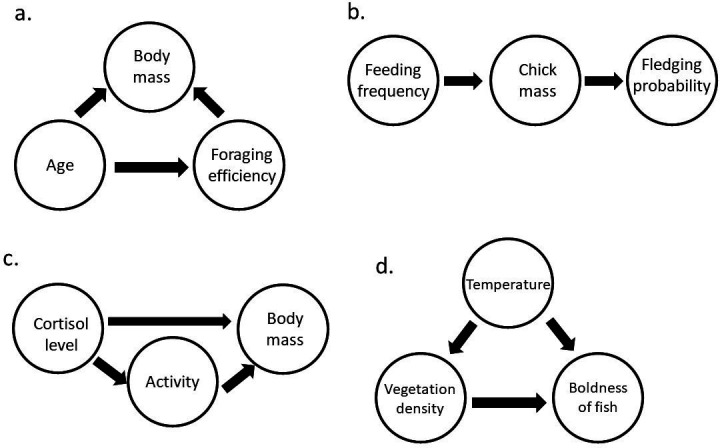
Four directed acyclic graphs showing collider, pipe and confounder variables. (a) A DAG for a study trying to estimate the effect of age on foraging efficiency, where body mass is a collider for this relationship. (b) A DAG for a study trying to estimate the effect of feeding frequency on fledging probability, where chick mass is a pipe variable. (c) A DAG for an experiment trying to study the effect of cortisol on body mass, where activity is a pipe variable. (d) A DAG for a field study estimating the impact of the density of vegetation (refuge) on the boldness of a fish, where the mean temperature influences the vegetation density as well as the boldness of fish (via metabolic rates), acting as a confounder.

Two ways in which adding control variables to a statistical model can wrongly influence estimates are commonly called colliders and pipes (figure 3) [9,18]. Collider variables are caused by both the predictor variable and the response variable (or caused by descendants of the predictor and response variables). There is no causal path through a collider between the arrows pointing into it (i.e. predictor and response variable both cause the collider variable and are not associated with each other through the collider, as it is downstream to both of them). However, by including such a collider variable in a statistical model, a path between the predictor and response variable is opened through the collider variable, thus creating an association between the predictor and the response variable. The direction in which the estimate is changed depends on the direction of the collider effect (i.e. the correlation will become more positive when the collider effect is positive, and more negative when the collider effect is negative). Hence, the inclusion of colliders sometimes causes significant results for associations that were absent in reality, or vice versa. For example, when a study tries to estimate the effect of age on foraging efficiency and the expected causal structure is like that in figure 3a. Here, both age and foraging efficiency independently affect body mass. It is important to not include body mass, because it is a collider. When body mass is added to the statistical regression model, a causal path between age and foraging efficiency is opened through body mass, therefore inflating (or deflating) the estimate of the effect of age on foraging efficiency. In sum, collider bias can decrease the accuracy of an estimated effect and can result in wrong conclusions about the strength and direction of this estimated effect.

Pipe variables are caused by the predictor variable and cause the response variable. They are also called mediators. For pipes, it is important to realise whether it is important for the research question to estimate the total effect of the predictor variable on the response variable, or the direct effect of the predictor variable on the response variable (see §2). When the total effect of the predictor variable should be estimated (e.g. in drug trials where the goal is to determine the effectiveness of a medicine), a pipe variable should not be conditioned on, as this would lead to false conclusions (e.g. about the effectiveness of a medicine). However, when a mechanism is studied and the influence of mediator variables (affected by the predictor variable) on the response variable is of interest, then pipe variables can be included. Yet, in such a case, it might be of interest to use path analyses or structural equation models (SEMs). Both types of models include a hypothesised causal network, and essentially incorporate multiple sub-models into one model. In other words, all arrows within a hypothesised causal network can be studied within a single model, and therefore more can be learned about how mechanistically an explanatory variable (directly and indirectly) can cause a response variable [19,20]. SEMs can additionally be extended so that latent variables (i.e. variables that cannot directly be measured) could be included in a model [9,21,22]. For example, when a study tries to understand the effect of feeding frequency on the probability that offspring fledge, and the causal structure is as in figure 3b, it depends on the biological question whether chick mass should be included in the statistical model, because chick mass is a pipe variable. When the goal is to estimate the total effect of feeding frequency on fledging probability, then chick mass should not be included. Yet, when the goal is to estimate only direct effects, then chick mass should be included. In this second case, a model will show no direct effect of feeding frequency, and a strong effect of chick mass on fledging probability. As another example, figure 3c shows a study that manipulated the cortisol levels in animals to estimate its effect on body mass (and we assume the causal structure as in figure 3c). The manipulation also changes all the intermediate variables, and hence, to estimate the total effect of the manipulation, including activity in a statistical model is wrong (it is a pipe), as it takes out the indirect effect of cortisol on body mass. However, when the experiment specifically wants to know how much of the change in the body mass is caused by a direct effect of cortisol, activity should be included in the statistical model. In such a case, it might be better to perform a path analysis, also including the direct effect of cortisol on activity, to ensure there is an effect of cortisol on activity.

Confounders, on the other hand, should always be included in the statistical model, and not including confounders may lead to spurious associations. Confounders causally affect both the response and the predictor variable. This means that there is a causal path between these variables through the confounding variable, unless the confounder is added to the statistical model. For example, figure 3d shows a DAG for a study testing the effect of vegetation density on boldness tendencies across populations in the field using measurements such as flight initiation distance. Temperature affects the vegetation densities, with higher temperature leading to denser vegetation, but also the boldness of the fish via their metabolic rates and therefore is a confounder. To estimate the effect of vegetation density on the boldness of fish, temperature should be included in the statistical model.

While the question of whether to include a variable in a statistical model is much simpler in experiments, it is nevertheless an important question for observational studies. Currently, many observational studies still hold the old belief that when two variables are not significantly correlated, they can both be included in a statistical model, in essence assuming that every variable in their DAG is completely independent and only affects the response variable. Yet, when being forced to articulate these assumptions, researchers might realise that effects are not independent. DAGs can thus be useful to aid the thought process and help in making hidden assumptions and relationships explicit. Moreover, assuming that many ecological variables are completely independent from each other seems illogical in field systems. We know that many factors in ecological systems affect each other (e.g. climatic effects might interact, a social environment can create non-independent data points across individuals), and in fact, we have dedicated whole research areas to it (e.g. systems ecology, community ecology). Additionally, the fact that two variables are only weakly (and potentially non-significantly) associated does not solve the issues that confounders, colliders and pipes cause. A weak collider variable could still inflate the estimated effect of the predictor variable on the response variable. Moreover, not including a confounder (which should correlate with the predictor and response variable) also decreases the accuracy of the effect in question. Hence, we would like to convince biologists that hypothesizing certain associations (with a DAG) is better than (implicitly) assuming that every variable is completely independent, unless it is explicitly hypothesized (preferably again with a DAG) that variables are independent from each other. This saves research from unnecessary inflations or deflations of estimates caused by colliders, pipes and confounders and thus makes our research better. Moreover, the addition of independent variables to models does not affect the accuracy of the estimation of the relationship in question, but only improves the precision of this estimate [18]. Whether that is necessary is up to the researcher, and might for example depend on how the data are analysed (e.g. Bayesian statistical results often already give information about precision).

### (b) DAGs to increase transparency in scientific research

Hypotheses in behavioural ecology are often broad and generalized, while we commonly test these hypotheses with much more specific and specialized (to one or a couple of species) statistical models. In this step from general to specific questions or hypotheses, numerous assumptions are made, usually based on the ecology of the study system. While researchers aspire to mention all these assumptions, it is easy to overlook some. This could stem from the fact that the ecological knowledge about a study system can seem trivial for researchers studying that system or from the fact that the complexity of a system can lead to certain assumptions being made implicitly or simply be overlooked. Yet, these underlying assumptions influence the expectations and also the outcome of a study. DAGs offer a simple graphical tool to clarify most of these underlying assumptions. This can decrease confusion among the readers and reviewers—and even the authors themselves—who often think about an overarching general hypothesis using the assumptions of the study system they work with/are familiar with.

**Figure 4.**
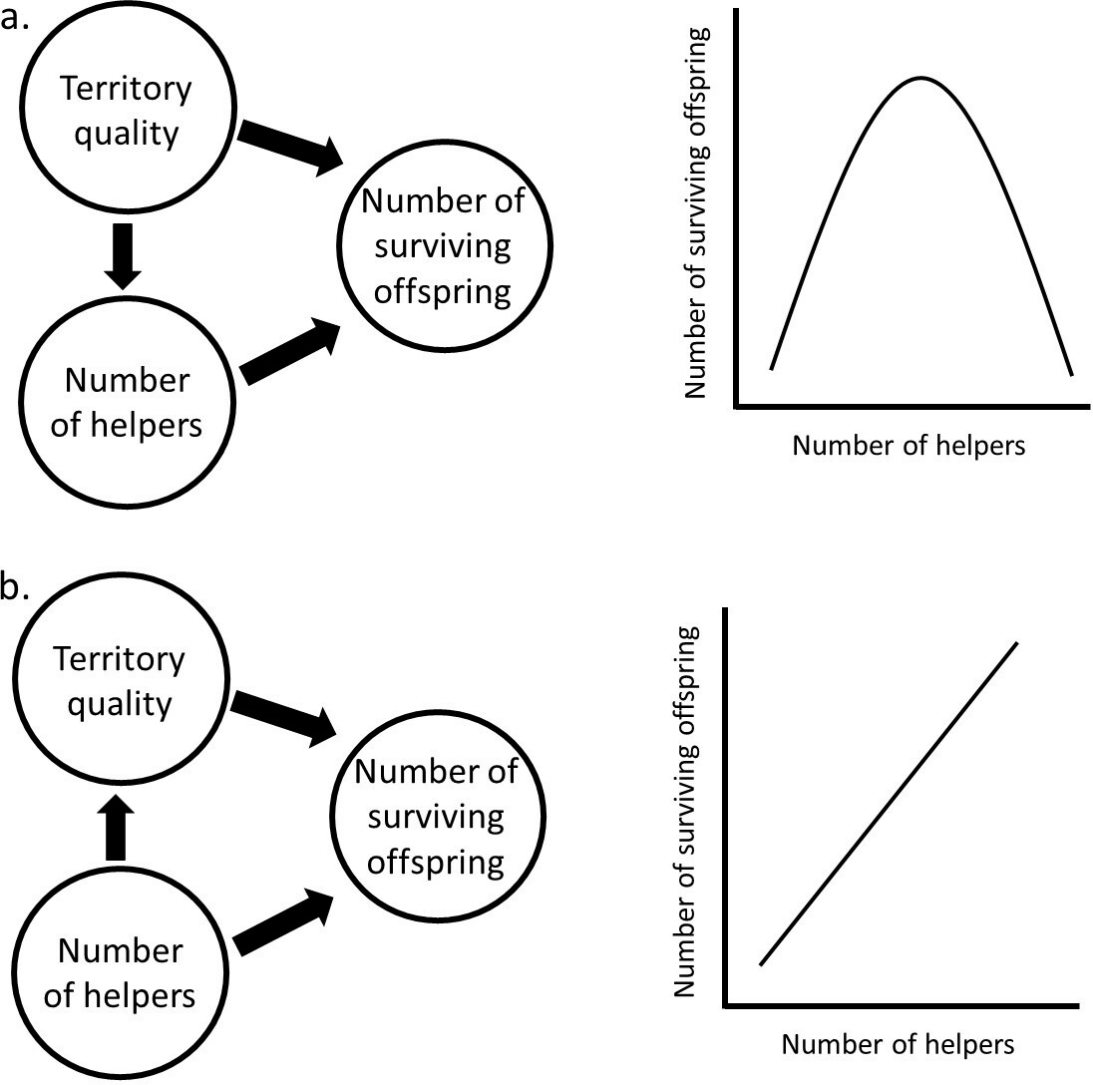
Two directed acyclic graphs (DAGs) about the effect of territory quality and number of helpers on the number of surviving offspring. (a) A case where territory quality causes the number of helpers. (b) A case where the number of helpers causes territory quality. Depending on the causal relationship between territory quality and number of helpers, the effect of number of helpers on number of surviving offspring changes.

For example, the evolution of cooperation and cooperative breeding is a well-studied topic in behavioural ecology [23], where the underlying ecological assumptions can have drastic effects on the evolutionary predictions (figure 4). In territorial species where territory size is more or less fixed, one would expect that acquiring helpers (subordinate individuals that help in raising offspring of dominant territory owners) depends on the territory quality. In other words, territory quality ‘causes’ the number of helpers (e.g. Seychelles warbler *Acrocephalus sechellensis* [24]; acorn woodpecker *Melanerpes formicivorus* [25]; see figure 4a). When territory quality is low, helpers might consume the few resources that are available, therefore leading to less resources for the offspring. When territory quality is high, there might be enough resources to sustain a certain number of helpers, but also for these helpers to increase feeding rates of offspring. In such species, we expect an optimal number of helpers per territory that depends on the quality of this territory, as there is a trade-off between the decreased resource availability due to helpers feeding themselves and the increased survival probability of offspring due to the assistance of these helpers. Alternatively, when helpers actively and substantially increase the size of the territory they reside on, and therefore increase the resource availability, helpers ‘cause’ the territory quality (e.g. cichlid *Neolamprologus obscurus* [26]; cichlid *N. pulcher* [27]; see figure 4b). In this case, helpers decrease the negative effect of them consuming resources, as they also assure extra availability of resources. Therefore, an increase in the number of helpers often has a positive effect on offspring survival, independent of the territory quality before a helper was present. These underlying ecological patterns are often clearly mentioned when studying the effect of helper presence on offspring survival/number of offspring, but are sometimes overlooked when studying more complex questions about cooperative breeding. Yet, they might still have a large impact on the expected evolutionary patterns. For example, over the last years, the question of whether cooperative breeding might buffer against harsh or unpredictable environments has received a lot of attention (e.g. [28–30]). This hypothesis could be consistent with study systems where the number of helpers influences territory quality, because in such a case, even in harsh environments, helpers might be able to improve territory quality enough for offspring to survive, while without helpers, this would not have been possible. Similarly, this hypothesis could be consistent with study systems where predation is the limiting factor of offspring survival instead of food availability, as in such a case, helpers might protect offspring from being preyed upon and increase their survival probability in that way, while there are enough resources for both helpers and offspring to consume. Yet, this buffering hypothesis seems illogical for species where the territory quality determines the number of helpers. In such a case, the competition for resources between helpers and offspring intensifies when conditions turn harsh, as there are now less resources available per individual. Hence, territories with fewer helpers might in fact produce more surviving offspring and thus becoming less social might buffer against harsh environments. When the expected outcomes depend on the ecology of the species, a DAG of the different study systems can clarify why cooperative breeding might buffer against harsh environments in certain cooperative breeding species, but not in others, as the arrow between territory quality and number of helpers points in the opposite direction in the two cases.

Another hypothetical example to illustrate that DAGs can be helpful to increase transparency is the effect of predator density on foraging behaviour and survival of prey (figure 2). While it is evident that the direct effect of an increase in predator density on the survival of individual prey is negative, this result might not be found or found to a different extent than expected when experimentally changing the predator density, because experiments manipulate the total effect of predator density on survival, instead of the direct effect. Predation could, for example, have indirect effects on survival because it might also influence the foraging efficiency of individuals, because prey might forage less when predators are around, furthering the negative effect of predation on survival. Moreover, predation could have a negative effect on the population density of prey, which might in fact increase the foraging efficiency of individual prey, as there is less competition between the remaining individuals, and thus potentially increase their survival. A DAG can show which factors are expected to affect foraging efficiency and survival and could also explain why it is not so evident to form an expectation about the total effect of predation on survival. In such cases, a DAG can clarify what the assumptions are underlying the effect of predation on survival, and can explain why different patterns can be found in different species, or in different populations within the same species. Furthermore, a DAG can show how different mechanisms could lead to the same result. Comparing a population with predators that hunt during the foraging period of prey with one who forages at a different time (e.g. nocturnal predators, see figure 2a,b), shows that the negative effect of predation on foraging efficiency can be different between different study systems. In the case of nocturnal predators, there is only a positive effect of predation on foraging efficiency (through density regulation). However, the effect of predation on survival for nocturnal predators can still be anything from strongly positive to strongly negative, depending on the strengths of the indirect prey density effect and the direct predation effect. As another example, there could be a difference in the population size of two populations of the same species (see figure 2a*,*c). In large populations, the effect of predation on the prey density (and especially competition for food between prey) is likely much smaller than in small populations. Therefore, there might be almost no positive effect of predation on foraging efficiency in large populations, but potentially a strong positive effect of predation on foraging efficiency in smaller populations. Hence, in large populations, it might be expected that predation only has a negative effect on survival, while in small populations, the effect of predation on survival might be anything from positive to negative. DAGs highlight these small differences between study systems in a succinct way, and make it clear to the reader what exactly is expected in a study, even when it is not clear whether the total effect will be positive or negative.

Using DAGs, thus makes it easier to follow exactly what the researcher is studying and how they expect the study system to work. This increases the readability and transparency of research for readers, and helps in improving the replicability of the (statistical) methods that were used. Next to that, DAGs can also help reviewers by adding clarity to their critiques. With a DAG, it is easier to separate whether authors have made a mistake in, or have a different opinion about, their statistical analysis or whether the reviewer and authors disagree conceptually on a question or its underlying assumptions, while in principle they agree on the statistical method. In our review of the 245 research articles [10], a model contained on average 6 variables (range = 2–23, median = 6). Given that there is a maximum of 15 causal arrows in a model with 6 variables, a misunderstanding about the exact hypothesis, including underlying assumptions, can easily happen. Incorporating a DAG can solve a lot of these issues. Moreover, in a recent report, 174 research teams were asked to analyse the same two datasets. The results in terms of effect sizes were strikingly variable, even presenting in opposite directions across research teams [31]. One of the main reasons was attributed to the substantial variation in the variables that were included as fixed and random effects in their statistical models. We believe that if researchers constructed a DAG prior to analysing their data, they would have a more principled approach to selecting variables for their analyses. This would shift the discussions towards why researchers had different underlying assumptions and their rationale for them. Instead of a crisis or caution about interpreting results in the field due to apparently valid statistical analyses leading to varied interpretations, the focus would be on the conceptual differences driving those choices. We argue that DAGs could thus improve the transparency about causal assumptions and the readability of an article, and with that potentially help solve the replication crisis, which is also facing this field [32,33]. When a DAG is included, it may become easier to replicate methodologies, including replicating the statistical decisions, which sometimes are not well described. In addition, it may become easier to conceptually replicate a study, where the same hypothesis or theory is tested in a different way (different contexts, different systems) to obtain generalizable results. DAGs aid this by identifying studies that incorporate the same underlying assumptions. Subsequently, DAGs can be used to distinguish studies with different underlying assumptions to start with and hence are not part of a conceptual replication (i.e. these studies where in fact not replicates, but researched a slightly different question, as shown in figure 4a,b). Moreover, DAGs can be a great addition to preregistrations, where they allow authors and peer reviewers to visualize the relationships clearer, leading to best methods for data analysis or take decisions on additional variables to measure. Ultimately, this helps reducing ‘research waste’ by enforcing better planning and reporting [34].

DAGs can also improve meta-analyses, as studies studying the same question but predicting the opposite given their underlying assumptions can be separated in a quantitative way, instead of combining studies to conclude apparent null effects. In recent times, meta-analysts in behavioural ecology suffer from the inability to delineate effects from complex statistical models, where it is not clear what variables are controlled for and why. This is especially true when the variables of interest are proxies (such as proxies of fitness, reproduction, parental care) or secondary variables that are not the response variable in the main statistical model, thus leading to the exclusion of studies. In this case, DAGs also provide a more targeted inclusion criterion, in addition to improving the transparency of studies for effective meta-analyses [35]. Lastly, DAGs can help new researchers (e.g. students or people changing fields) to understand the key differences between study systems that are important for the questions they wish to study, without having to spend considerable amounts of time researching the ecology of all these species.

### (c) Where to start?

So far, we have provided examples, outlined basic structures and discussed how DAGs can make scientific discourse efficient and transparent. However, constructing a DAG is entirely based upon the expert subject knowledge of the researchers. After identifying the research question(s) and defining the estimand, it is important to include those relevant variables— the predictor and the response variables. Next, it is important to include all the common causes of our predictor and response variables. When introducing a new variable, its common causes must also be included. It is important to keep in mind that DAGs are not meant to be realistic depictions of your study system; rather, they aim to succinctly represent the research question and outline the underlying hypotheses. Consequently, mediators or mechanisms should only be included if they directly pertain to the research inquiries. For the keen reader or enthusiastic adopter of DAGs, we recommend referring to key literature on DAGs and causal inference. On tips how to build a DAG and use causal inference, we would like to refer to Laubach *et al.* [36], and Arif & MacNeil [11] for explanations with biological examples, to McElreath [9] (including the accompanying YouTube videos) for explanations on causal inference especially well-suited to beginners, and to Judea Pearl’s work (e.g. [7,8,18,22,37]) for a more in depth understanding.

Drawing DAGs can be done by hand, but there is also an R package [38] and website (https://www.dagitty.net/dags.html[) available to help with this. The R package also aids in identifying the conditional independencies and adjustment sets, to help with which variables to condition for if your DAG is complex.

We would like to advise readers to draw DAGs before conducting their research (as it is a formalised version of their hypothesis), and they could be included in preregistrations if researchers like to do so. Moreover, while we think DAGs are valuable additions to manuscripts, we understand that their inclusion in a manuscript might be limited by length or figure restrictions, and we encourage researchers to include a DAG in their electronic supplementary materials in such cases.

### (d) Limitations of DAGs

DAGs are useful and simple representations of causal assumptions, and we hope to have convinced the readers of their strengths by now. But they can come with a few drawbacks when put into practical use. First, systems in behavioural ecology are rarely simple, hence DAGs can become complex quite easily (but see [39] for an example of a more complex DAG). Decisions about the causal relationships in the DAG have to be made (e.g. should there be an arrow between two variables, and if so, in which direction), and it can be difficult to pick the ‘right’ answer. Indeed, there is no one true DAG, unless all causal relationships are well-known. However, in essence, this is an issue of knowledge about the system, and not one of drawing DAGs *per se*. In such cases, DAGs might help uncover which assumptions are still unknown and should be tested. In case of multiple potential alternative hypotheses or assumptions, the most likely DAG may be chosen, and the alternative hypotheses/DAGs can be discussed. Moreover, if a causal pathway is unknown, we argue that it is still better to have a hypothesis about it (which consequently can be tested or critiqued) than to implicitly have an underlying assumption about a relationship (or lack thereof) between variables.

Second, DAGs still do not convey all information about the statistical analyses. For instance, DAGs cannot help make a decision or convey information about the following: Should variables be added as fixed or random effects? Is the expected relationship linear, quadratic or another shape, and is the relationship positive or negative? To summarize all this information, SCMs can be used, which are mathematical representations of a statistical model. Yet, some biologists in behavioural ecology tend to be a bit averse to math [40], and hence we argue that using a visual representation (a DAG) is a good start to transparently communicate causal assumptions in statistical models. Moreover, some of this information could potentially be added to DAGs, such as using colour coding or different shapes (e.g. green lines for hypothesized positive relationships, red lines for hypothesized negative relationships; random effects written in italics or contained in a square box instead of in a circle).

Third, causal inference and, therefore, to some extent DAGs, are unable to deal with interaction effects, where the effect of a variable is not on an outcome variable, but on the relationship between two variables. Some have suggested representing this by making an arrow point to another arrow (e.g. [41,42]), but the theory around the meaning of interactions in terms of causality is not as well-developed as DAGs.

Last, DAGs are acyclical, which means that causal inference so far is unable to include cyclical interactions or reciprocity. Yet again, we would argue that being unable to disentangle feedback loops between cause and effect is an inherent problem in many types of science and is not an issue of DAGs in itself. In some cases, time series analyses could help to disentangle these reciprocal interactions, if it is possible to find a time frame where *x* solely affects *y*, and another time frame where *y* solely affects *x* (e.g. timing of migration might cause timing of breeding, and timing of breeding might cause the timing of migration in the next year). In such a case, two DAGs could be produced for each time frame and these hypotheses could accordingly be tested in the correct time frames. However, when it is not possible to disentangle reciprocal interactions, DAGs cannot help in solving this issue. In such cases, researchers will have to decide which direction is more important for their question, and be cautious with interpreting their results.

## 4. Conclusion

With this paper, we would like to convince the reader that using DAGs in behavioural ecology and beyond is beneficial. DAGs increase the readability of papers because they show underlying assumptions that are not always mentioned. Exposing these underlying assumptions increases the transparency of research. DAGs can stand the test of time: even if statistical tools undergo changes or advances such that reading a model description to work out causal assumptions becomes more challenging, DAGs can offer a simple reporting standard and a common language for causal assumptions that justify statistical models. Thus, DAGs could contribute to solving the replication crisis and make the work of reviewers and researchers doing meta-analyses easier and more rigorous. Additionally, DAGs are extremely useful for considering which variables should be included in statistical models. Moreover, we think that a hypothesized causal structure is valuable (which often will be thought through when a DAG is produced), because in our opinion, they are always better than implicitly hypothesizing without adequate thought that every variable in a model is completely independent. DAGs, just as hypotheses, might be wrong, but by showing a DAG, these mistakes are easier to find, and at least statistical mistakes caused by expected colliders, pipes or confounders can be prevented .

## Data Availability

Data and data collection procedure are available openly on a Zenodo repository [10].
